# Assessment of a randomized controlled trial on the safety of pre-placing bronchial balloons in transbronchial lung cryobiopsy for diagnosing interstitial lung disease

**DOI:** 10.1186/s40001-024-01871-y

**Published:** 2024-05-03

**Authors:** Yiding Bian, Guowu Zhou, Qian Gao, Mingming Deng, Run Tong, Yang Xia, Jieru Lin, Gang Hou, Huaping Dai

**Affiliations:** 1 National Center for Respiratory Medicine, State Key Laboratory of Respiratory Health and Multimorbidity, National Clinical Research Center for Respiratory Diseases, Institute of Respiratory Medicine, Chinese Academy of Medical Sciences, Beijing, China; 2 Department of Pulmonary and Critical Care Medicine, Center of Respiratory Medicine, China–Japan Friendship Hospital, 2 Yinghuayuan East Street, Chaoyang District, Beijing, 100029 China; 3 Chinese Academy of Medical Sciences, Peking Union Medical College, Beijing, 100730 China; 4 Department of Respiratory and Critical Care Medicine, Key Laboratory of Respiratory Disease of Zhejiang Province, Second Affiliated Hospital of Zhejiang University School of Medicine, Hangzhou, 310052 Zhejiang China; 5 Department of Respiratory and Critical Care Medicine, Guizhou Provincial People’s Hospital, Guiyang, 550002 China

**Keywords:** Transbronchial lung cryobiopsy, Pre-placed bronchial balloon, Interstitial lung disease

## Abstract

**Rationale and objectives:**

Bleeding is a major complication of transbronchial lung cryobiopsy (TBLC), and pre-placing a bronchial balloon is one of the clinical practices used to prevent it, but with very weak evidence, which should be confirmed. This study aimed to conduct whether pre-placing a bronchial balloon in TBLC for diagnosing interstitial lung disease (ILD) is more safety.

**Materials and methods:**

In this prospective, single-center, randomized controlled trial, patients with suspected ILD were enrolled and randomly assigned to pre-placed balloon and none-pre-placed balloon groups. The primary outcome was incidence of moderate bleeding in each group. The secondary endpoints were the incidence of severe bleeding, pneumothorax, and other procedural complications.

**Results:**

Exactly 250 patients were enrolled between August 2019 and March 2022, with 125 in each group. There were no significant differences in severe bleeding between the none-pre-placed balloon group and pre-placed balloon group (1.6% vs. 0.8%; adjusted *p* = 0.520), while more moderate bleeding occurred in the none-pre-placed balloon group (26.4% vs. 6.4%, adjusted *p* = 0.001), as well as more use of hemostatic drug (28.0% vs. 6.4%, adjusted* p* = 0.001). Three patients in the none-pre-placed balloon group used the bronchial balloon. More samples could be acquired in the pre-placed balloon group than in the none-pre-placed balloon group (3.8 ± 0.9 vs. 3.1 ± 0.9, *p* < 0.001). There were no significant differences in multidisciplinary discussion (MDD) between the two groups (89.6% vs. 91.2%, adjusted *p* = 0.182).

**Conclusion:**

A pre-placed bronchial balloon can reduce the incidence of moderate bleeding and increase the confidence of the bronchoscopists. However, it had no effect on increasing the diagnostic rate of MDD and reducing severe bleeding.

*Registration number*: NCT04047667 (www.clinicaltrials.gov identifier).

**Supplementary Information:**

The online version contains supplementary material available at 10.1186/s40001-024-01871-y.

## Introduction

Interstitial lung diseases (ILD) are a heterogeneous group of disorders comprising more than 200 entities [1]. Although the clinical manifestations of ILD are similar in different entities, the corresponding therapeutic options differ, as well as their prognosis [2–4]. Therefore, there remains a need for precise and specific diagnostic methods to guide clinical management of this complex condition. In the current clinical practice, a reliable diagnosis of ILD can be established in the majority of cases by patient history, imaging and serologic analysis, followed by multidisciplinary discussion (MDD). However, in ~ 10–30% of cases, ILD cannot be accurately classified, and therefore, invasive procedures for tissue collection are required to make specific pathologic diagnoses for definitive classification [5, 6]. Surgical lung biopsy (SLB) has long been the preferred modality for tissue sampling because, in the majority of cases, traditional transbronchial forceps biopsies provide tissue samples that do not allow for reliable pathologic diagnosis [7, 8]. But along with a high diagnostic rate comes the fact that SLB is associated with significant morbidity and mortality [9–11], especially in those patients with comorbidities or with severely impaired lung function. At the same time, not all medical centers have the capacity to perform SLB.

Recently, transbronchial frozen biopsy (TBLC) has been proposed as an alternative modality for tissue sampling in patients with suspicious ILD [7, 12–14]. It is increasingly being used for assessing ILDs because it provides tissue samples with a higher percentage of alveolar tissue and fewer crush artifacts than conventional transbronchial biopsies [15]. The diagnostic value of TBLC for ILD is comparable to that of surgical lung biopsy (SLB) but less invasive [16, 17]. A conditional recommendation was made to regard TBLC as an acceptable alternative to SLB in centers with appropriate expertise [18]. TBLC is recommended for the diagnosis of hypersensitivity pneumonitis (HP) [19] and idiopathic pulmonary fibrosis (IPF) [20]. Therefore, TBLC is increasingly being recognized as a potential alternative to SLB for the diagnosis of ILD.

Meanwhile, it has been reported that ~ 0.2–56.4% of moderate bleeding [13, 21–23] and 0.6–21.1% of pneumothorax [22, 24–26] occurred during TBLC; thus, TBLC is still an invasive diagnostic method that may pose a threat to patients [27, 28]. To avoid life-threatening bleeding, some centers routinely pre-place bronchial balloons during TBLC procedures to ensure safety, as the 2020 CHEST Guideline and Expert Panel Report recommended that TBLC be performed with bronchial blockers either through an endotracheal tube or a rigid bronchoscope [27]. However, this was an ungraded consensus-based statement. At the same time, bronchial balloon placement may be time consuming and increase medical costs.

To our knowledge, no study has prospectively investigated whether it is useful to pre-place a bronchial balloon before TBLC to reduce the risk of bleeding and other compliments. Therefore, we conducted this study to discuss the safety of TBLC in patients with ILD, using a pre-placed bronchial balloon or not.

## Methods

This was a prospective, single-center, randomized controlled trial that enrolled patients with suspected ILD at the China-Japan Friendship Hospital.

### Patients

Each patient suspended ILD underwent a first MDD after hospitalization which also determined whether a TBLC procedure was needed. Patients who met the following eligibility criteria were recommended to undergo TBLC: > 18 years of age; a diagnosis of ILD could not be established after integration of clinical features: forced vital capacity (FVC) > 50% and diffusing capacity of the lung for carbon monoxide (DLCO) > 35%. Patients who met the following criteria were excluded: acute exacerbation in the past 30 days, bleeding diathesis, anticoagulant therapy, current use of antiplatelet drugs, severe pulmonary hypertension (with estimated right ventricular systolic pressure > 40 mmHg or signs of right ventricular dysfunction on echocardiogram), severe respiratory failure, severe liver or kidney dysfunction, severe cardiac insufficiency, or platelet count < 50 × 10^9^/L. This trial was approved by the ethics committee of the China–Japan Friendship Hospital (2017-25-1) and was registered at www.clinicaltrials.gov identifier (NCT04047667).

### Study design, randomization, and blinding

The number of cases was calculated based on the estimated incidence of moderate bleeding for the primary endpoint. The estimated moderate bleeding rate was 20% according to the previous study [10]. Non-inferiority tests for differences between two proportions were used to estimate the sample size using PASS 11. We used a 0.15 non-inferiority difference with 0.8 power and the α was 0.025, so we needed to recruit 112 patients in each group. Considering 10% loss to follow-up, a total 124 patients should be needed for each group (Additional file 1: Fig. S1), and finally 250 patients were enrolled in the final analysis. All patients included in this study signed written informed consent before enrollment.

**Fig. 1:**
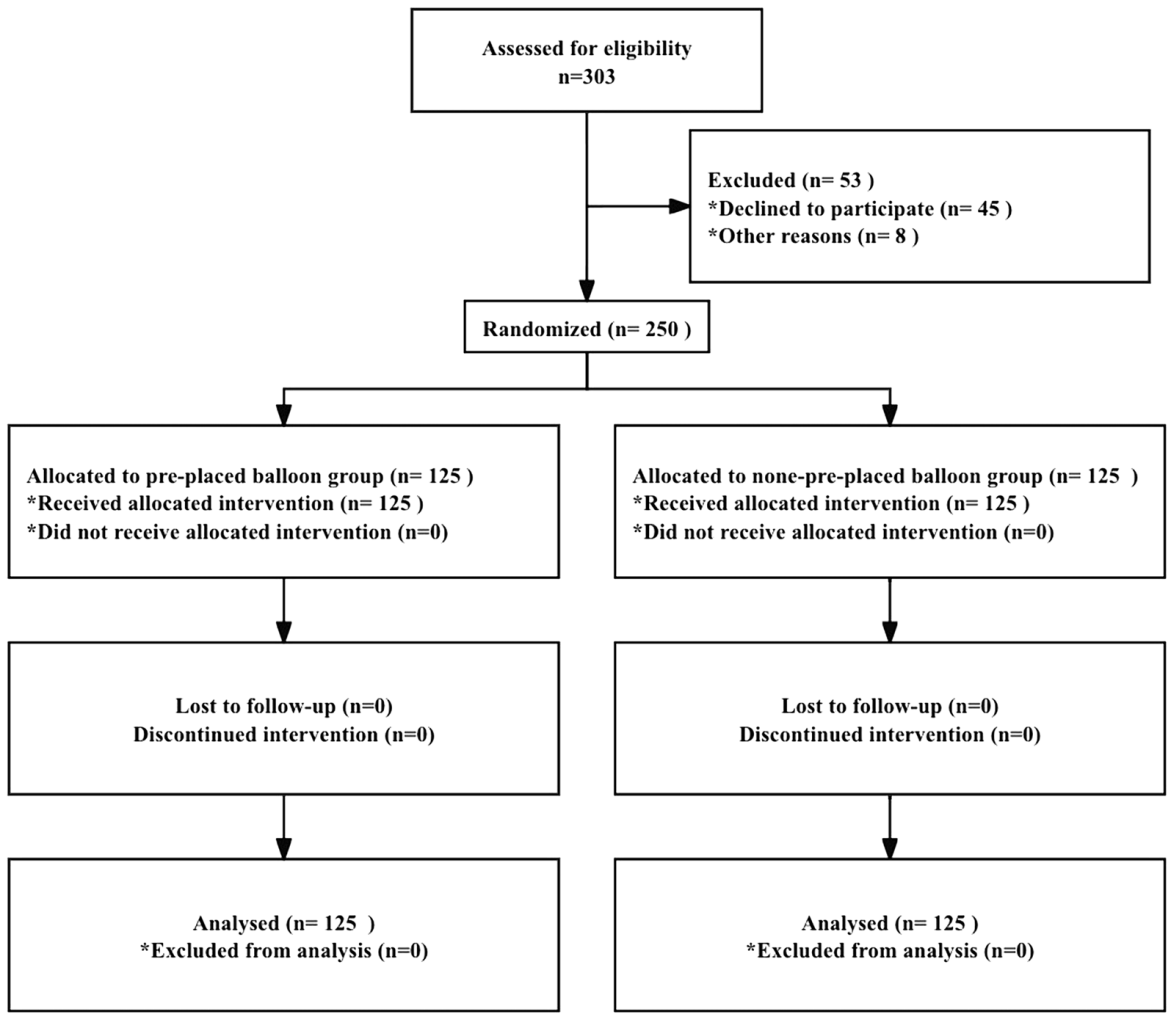
Flowchart of patients enrolled in this study

The participants were randomly assigned to two groups (pre-placed balloon group and none-pre-placed balloon group) according to a computer-generated random number list by a research assistant. Blinding of patients and clinical teams was not feasible because of the nature of the intervention. Only the pathologists were blind to the study assignment throughout this study (Fig. 1).

### Endpoints

The primary endpoint was the incidence of moderate bleeding of patients in the pre-placed balloon group and none-pre-placed balloon group. The following secondary endpoints were also evaluated: the incidence of severe bleeding, the incidence of pneumothorax and the possible subsequent closed thoracic drainage, and the incidence of postoperative infection, postoperative hospital stay, use of hemostatic drugs, mortality, and diagnostic rate of MDD of patients in both two groups.

Proposed scale for assessing severity of bleeding during transbronchial biopsy was defined as the previous study [29]: Grade 1: Requiring less than 1 min of suctioning or wedging of the bronchoscope resulting in spontaneous cessation of bleeding; Grade 2: Suctioning more than 1 min or need for rewedging of the bronchoscope or instillation of cold saline, vasoactive substances, or thrombogenic agents; Grade 3: Selective intubation with endotracheal tube or balloon/bronchial blocker for less than 20 min or premature interruption of the procedure; Grade 4: Persistent selective intubation > 20 min or new admission to the intensive care unit or packed red blood cell transfusion or need for bronchial artery embolization or resuscitation. Grade 1 was regarded as mild bleeding. Grade 2 was regarded as moderate bleeding. Grade 3 and Grade 4 were regarded as severe bleeding.

Postoperative hospital stay was defined as the number of days from the day of operation to the day of discharge. Mortality was defined as death occurring within 7 days after TBLC.

### TBLC procedure

Bronchoscopy was performed under general anesthesia using a rigid bronchoscope (Karl Storz, Germany). A cryoprobe (ERBE; Solingen, Germany) was advanced as far as possible through the working channel of the bronchoscope to the target bronchial segment. Moreover, 1.9-mm probes were used in all patients, as recommended by the CHEST Guideline and Expert Panel Report [27]. The cryoprobe was then retracted to 1 cm, and the bronchoscope was secured to the holder. X-rays (Artis Zee III ceiling, Siemens AG, Munich, Germany) were then performed to assess the location of the cryoprobe (~ 1–2 cm from the pleural surface). If necessary, the cryoprobe should be repositioned to ensure that it is ~ 1 cm from the pleura. TBLC was performed after probe positioning using carbon dioxide as a cryogen for 6–8 s. In the pre-placed balloon group, a bronchial balloon blocker (CRE balloon, Boston Scientific Microvasive, Natick, MA, USA) was pre-placed through the working channel and immediately filled after the completion of TBLC to stop bleeding. In the none-pre-placed balloon group, a bronchial balloon was prepared in the bronchoscope room. It would not be placed unless visible bleeding was found and procedures were useless including suctioning, wedging of the bronchoscope, rewedging of the bronchoscope or instillation of cold saline, vasoactive substances, or thrombogenic agents. Further hemostatic treatment (selective intubation with endotracheal tube or balloon/bronchial blocker or new admission to the intensive care unit or packed red blood cell transfusion or need for bronchial artery embolization or resuscitation) would be used in order of severity. Postoperative chest X-rays in the following 1 h were used to screen for pneumothorax. Following the intervention, patients were managed with routine postoperative care and were followed until discharged.

### Statistical analysis

This study was designed to test the hypothesis that the probability of severe bleeding in the none-pre-placed balloon group is not higher than that in the pre-placed balloon group in TBLC. We presented standard descriptive statistics, with categorical variables presented as percentages and continuous variables as means and standard deviations. Comparisons between groups of categorical data were performed using Fisher’s exact test or Pearson’s Chi-square test. Continuous variables were assessed for normal distribution, and comparisons were conducted using t-tests. The adjusted endpoints of the two groups and their differences were calculated using logistic regression for categorical variables and liner regression for continuous variables after adjusting for the factors of the TBLC procedure (adjusted byd number of samples and sample size). All *p*-values were two sided, with *p* < 0.05 indicating statistical significance. All data were analyzed using Stata SE 15.1 (StataCorp, College Station, TX, USA).

## Results

A total of 250 patients were enrolled from August 2019 to March 2022 and divided into a pre-placed balloon group (*n* = 125) and a none-pre-placed balloon group (*n* = 125). The mean age was 56.5 ± 12.5 years. 126 males and 124 females were included in this study. The baseline characteristics between the two groups are shown in Table 1.

**Table 1:** Baseline characteristics

	Pre-placed balloon *n* = 125	None-pre-placed balloon *n* = 125
Male	62 (49.6)	64 (51.2)
Age, y	57.5 ± 12.9	55.5 ± 12.1
Smoking status		
Never	85 (68.0)	78 (62.4)
Current	17 (13.6)	15 (12.0)
Former	23 (18.4)	32 (25.6)
Lung function measurements, median (IQR)		
FVC, %pre	87.5 (74.8–101.2)	86.0 (71.5–99.3)
DLCO, %pre	65.4 (56.5–79.2)	64.9 (56.2–76.0)
Comorbid conditions		
PAH	0 (0.0)	1 (0.8)
Hypertension	28 (22.4)	27 (21.6)
CHD	11 (8.8)	5 (4.0)
Arrhythmia	1 (0.8)	2 (1.6)
Hepatitis	2 (1.6)	2 (1.6)
Hypohepatia	0 (0.0)	1 (0.8)

*FVC*  forced vital capacity, *DLCO* diffusion capacity of the lung for CO, *PAH* pulmonary arterial hypertension, *CHD* coronary heart disease

Data are presented as numbers (%) unless indicated otherwise

The number of samples was 3.8 ± 0.9 vs. 3.1 ± 0.9 (*p* < 0.001) between the pre-placed balloon group and the none-pre-placed balloon group, while the sample size was 21.4 ± 9.0 vs. 24.2 ± 8.3 (*p* = 0.009) (Table 2). The procedure duration of pre-placed balloon group was significantly longer than that of none-pre-placed balloon group (28.0 ± 5.8 vs. 15.3 ± 3.3, *p* < 0.001). There were no differences in number of lung lobes between the two groups.

**Table 2:** Biopsy data of TBLC

	Pre-placed balloon *n* = 125	None-pre-placed balloon *n* = 125	*p*-value
Number of samples	3.8 ± 0.9	3.1 ± 0.9	< 0.001
Sample size, mm^2^	21.4 ± 9.0	24.2 ± 8.3	0.009
Procedure duration, min	28.0 ± 5.8	15.3 ± 3.3	< 0.001
Number of lung lobes, *n* (%)			1.000
Single lung lobes	123 (98.4)	123 (98.4)	
Multiply lung lobes	2 (1.6)	2 (1.6)	

*TBLC* transbronchial lung cryobiopsy

There were no significant differences in severe bleeding between the pre-placed balloon group and the none-pre-placed balloon group (0.8% vs. 1.6%; adjusted *p* = 0.520), while more moderate bleeding occurred in the none-pre-placed balloon group (6.4% vs. 26.4%, adjusted *p* = 0.001). Three patients in the none-pre-placed balloon group used the bronchial balloon to stop bleeding. There were no significantly differences of hospitalization time after the procedure between the two groups (6.7 ± 4.2 vs. 7.3 ± 4.0; adjusted *p* = 0.122). There were no significant differences in pneumothorax (0% vs. 0%), and postoperative infection (6.4% vs. 4.0%; adjusted *p* = 0.931) between the two groups. The none-pre-placed balloon group used more hemostatic drugs than the pre-placed balloon group (28.0% vs. 6.4%; adjusted *p* = 0.001) (Table 3).

**Table 3:** Outcomes and complications of TBLC

	Pre-placed balloon *n* = 125	None-pre-placed balloon *n* = 125	*p*-value	Adjusted* p*-value*
Postoperative hospital stay, d	6.7 ± 4.2	7.3 ± 4.0	0.228	0.122
Bleeding			< 0.001	0.001
Grade 1	116 (92.8)	90 (72.0)		
Grade 2	8 (6.4)	33 (26.4)	< 0.001^†^	0.001^†^
Grade 3	1 (0.8)	2 (1.6)		
Use of hemostatic drug	8 (6.4)	35 (28.0)	< 0.001	0.001
Pneumothorax	0 (0.0)	0 (0.0)	–	–
Postoperative infection,	8 (6.4)	5 (4.0)	0.393	0.931
Mortality	0 (0.0)	0 (0.0)	–	–
MDD confirmed, *n* (%)	112(89.6)	114 (91.2)	0.668	0.182

*TBLC* transbronchial lung cryobiopsy

^*^Adjusted for number of samples, sample size, and procedure duration

^†^We combined mild bleeding and severe bleeding and compared the difference in the incidence of moderate bleeding between the pre-placed balloon group and none-pre-placed balloon group

Data are presented as numbers (%) unless indicated otherwise

Between the pre-placed balloon group and the none-pre-placed balloon group, MDD diagnostic rates were 89.6% and 91.2%, respectively (adjusted *p* = 0.182). Connective tissue disease-related ILD (CTD-ILD) (20.0%, 50/250), non-specific interstitial pneumonia (NSIP) (16.0%, 40/250), granulomatous lung disease (14.8%, 37/250), and IPF (10.4%, 26/250) were the most common MDD diagnosis in this study, which were following with special forms of ILD (5.2%, 13/250), desquamative interstitial pneumonia (DIP) (3.6%, 9/250), respiratory bronchiolitis-ILD (RB-ILD) (302%, 8/250), cryptogenic organizing pneumonia (COP) (2.4%, 6/250), and so on (Table 4).

**Table 4:** MDD pattern on basis of TBLC

Categories and subcategories	Pre-placed balloon *n* = 125	None-pre-placed balloon *n* = 125
Secondary ILD of known etiology		
Connective tissue disease-related ILD (CTD-ILD)	21	29
Drug or toxicant related ILD	0	4
Occupational or environmental related ILD	1	1
Granulomatous lung disease	23	14
Special forms of ILD	4	9
Idiopathic interstitial pneumonia (IIP)		
Idiopathic pulmonary fibrosis (IPF)	15	11
Non-specific interstitial pneumonia (NSIP)	22	18
Respiratory bronchiolitis—ILD (RB-ILD)	5	3
Desquamative interstitial pneumonia (DIP)	2	7
Cryptogenic organizing pneumonia (COP)	4	2
Idiopathic lymphocytic interstitial pneumonia (ILIP)	1	1
Unclassified ILD	2	4
Tumor	6	6
Infection	4	2
Others	2	3
Unconfirmed	13	11

*MDD* multidisciplinary discussion, *TBLC* transbronchial lung cryobiopsy, *ILD* interstitial lung disease

Special forms of ILD included pulmonary alveolar proteinosis, lymphangioleiomyomatosis, pulmonary Langerhans cell histiocytosis, and so on

## Discussion

Increasing evidence has proven the diagnostic value of TBLC for various diseases. In particular, the American Thorax Society has incorporated TBLC into the diagnostic guidelines for HP and IPF, and its value in the clinical diagnosis of ILD has also increased [18, 20, 27, 30]. Recent large-scale prospective, multicenter, randomized controlled study studies have also demonstrated good agreement between TBLC and SLB obtained sequentially in the same patients, supporting the clinical utility of TBLC as an alternative to SLB for patients requiring lung tissue for interstitial lung disease diagnosis. However, with the recognition of TBLC diagnosis of ILD, its security must also be considered. However, as TBLC becomes recognized for the diagnosis of ILD, its safety must also be taken into consideration. TBLC is, after all, an invasive operation, and bleeding is its single most common complication. Currently in guidelines and expert consensus, the use of bronchial balloons is recommended for TBLC procedures, but there is no high-level research evidence to validate this. To the best of our limited knowledge, our study is the first prospective randomized controlled study to explore this, and our results suggest that pre-positioned bronchial balloons better prevent moderate bleeding and are effective in increasing bronchoscopists’ confidence in the operation, but have no effect on the final MDD diagnosis rate or the rate of severe bleeding.

Bleeding was the most common complications inevitably caused by TBLC. In a meta-analysis of 12 studies [22], moderate bleeding after TBLC was observed in 65 of the 383 patients (16.9%). A recent meta-analysis demonstrated that any bleeding (mild through severe) in 39% (95% CI 23–55%) of patients, but the analysis was limited by inconsistent estimates across trials. When the meta-analysis was repeated using only studies that reported using a bronchial blocker, the bleeding rate was 24% (95% CI 14–34%) [14]. The overall incidence of moderate bleeding and severe bleeding in our study was 17.2%, which was similar to the findings of previous studies. Furthermore, our study showed that there was no difference in the risk of severe bleeding caused by TBLC between the groups with pre-placed balloon and none-pre-placed balloon, which was truly a mortal threat. However, severe bleeding is, after all, a very rare complication that when it occurs is a serious threat to the patient's life. In our study, only one patient in the pre-placed balloon group experienced severe bleeding, compared to 2 cases in the none-pre-placed balloon group. Though not statistically significant, it was double in terms of incidence. Therefore, we believe that pre-placing of the balloon to prevent severe bleeding is worthwhile. It has also been reported in the literature that life-threatening complications may occur if the pre-placed balloon is not used to prevent severe bleeding [31]. However, we believe that the use of X-rays to ensure that the probe is in the periphery of the lung is an important safeguard against severe bleeding. Therefore, a pre-placed balloon may not be an essential element in reducing severe bleeding. As the study showed that, given the rarity of events, the aggregated frequency of severe bleeding was negligible (0%, 95% CI 0–0%) in patients undergoing TBLC [14]. The choice of operating sites and areas that need to be avoided to prevent severe bleeding is deeply considered, which may be critical. Moreover, we need to pay attention to the role of navigation during the operation. Nevertheless, our study also found that pre-placement of a bronchial balloon significantly reduced the risk of moderate bleeding, which is the most common complication in TBLC. The recent meta-analysis demonstrated that the rate of moderate bleeding of TBLC was 11.7% (95% CI 9.1–14.9%) [32]. Given the relatively higher rate of moderate bleeding, pre-placed balloons seem to be a better choice to ensure the patients’ safety. In addition to this, the size of the cryoprobe may also be a factor in bleeding, but there is no high-quality evidence to suggest this. Currently, there are cryoprobes including 2.4-mm, 1.9-mm, 1.7-mm, and 1.1-mm. Two retrospective studies and one prospective study found no statistically significant difference in the incidence of moderate bleeding between the 2.4-mm and 1.9-mm cryoprobes, although the incidence of moderate bleeding was somewhat higher with the 2.4-mm cryoprobe. A small randomized controlled study also showed no significant difference in the incidence of moderate bleeding between the 1.7-mm and 1.9-mm cryoprobes. So, it is reasonable that attempting freezing from the shortest time may be a wonderful way to minimize bleeding from large cryoprobes.

Pneumothorax is another serious life-threatening complication of TBLC. No pneumothorax occurred in our study, which is significantly lower than the incidence of pneumothorax reported in the literature [22]. A previous study at our center found that the incidence of pneumothorax was only 1.9% [33], which is similar to the findings of this study. Confirming the proper distance between the probe and pleura before TBLC is an important measure to reduce pneumothorax. Furthermore, it has been reported that the risk of pneumothorax is probably related to the size of the probe and the method of guidance. Previous studies showed that the risk of pneumothorax increased with the use of 2.4-mm probes compared to 1.9-mm probes [34, 35], while Zhou’s study [36] showed that there was no difference in the risk of pneumothorax owing to the probe size, but guided by cone-beam computed tomography (CBCT), which is attributed to the low prevalence of pneumothorax. A recent study also showed that there was no significant difference in occurrence of pneumothorax between the 1.7-mm cryoprobe and 1.9-mm cryoprobe [37]. Therefore, probe size and guidance may affect the risk of pneumothorax (not the only effect) rather than balloon pre-placement. At the same time, there was no significant differences in our study in terms of postoperative infections, probably because the incidence of such conditions itself was low and the bronchial balloon was mainly targeted for the prevention of bleeding.

At the same time, bronchoscopists obtained more samples in the pre-placed balloon group, indicating that the pre-placed bronchial balloon allows the operator to sample with higher confidence when performing TBLC, although the pre-placed balloon group did not have a higher rate of final MDD diagnosis. According to previous studies, the diagnostic yield of TBLC was influenced by the number of samples taken and the number of biopsy sites. It was significantly increased when at least 2 samples were obtained from 2 different sites (either in the same lobe or in different lobes) [38]. Moreover, the size of cryoprobe may influence the diagnosis yield of TBLC. Previous study demonstrated sample acquired by cryoprobe with larger size had better sample quality. Although there were no differences in the final MDD diagnostic rate between probes with different sizes [36, 37], probe size and specimen quality may be points to consider. However, pre-placing the bronchial balloon significantly prolongs the operating time of the procedure. This may complicate an inherently simple operation, and the increased operating time also increases the risk of the operation to some extent. At the same time, it is important to note that a bronchial balloon is relatively expensive to spend at our center, as well as at other centers of China. If every patient is pre-placed with bronchial balloons, the cost-effectiveness is a problem worth considering. Of course, the problem varies greatly, depending on the country and region. In conclusion, pre-placing a bronchial balloon can increase the bronchoscopist’s confidence in the maneuver but may not be of great benefit to the final MDD diagnosis.

Our study had some limitations. Firstly, this was a single-center study that may represent only the practical level and experience of our center. Different medical centers may have different experiences and practice processes in terms of TBLC operation experience. The external validity of this study therefore needs to be considered with caution, and therefore, future larger multi-neutral randomized controlled trials need to be conducted for validation. However, over 200 TBLCs were conducted in our center each year, which was a relatively large amount and may provide some evidence. As far as procedure of TBLC is concerned, the bronchoscopists in our center may be relatively more experienced. Secondly, the relatively good pulmonary function of the patients in our study who underwent TBLC may also account for our lower bleeding rate and associated complications. Further studies are needed in the future to include critically ill patients for safety evaluation, to go for a refined exploration whether pre-placed bronchial balloon has greater benefits for safety of TBLC. Thirdly, we only used rigid bronchoscopy, and TBLC under tracheal intubation could not be performed; further study should be conducted to improve the process of TBLC under tracheal intubation.

Altogether, our study showed that pre-placing a bronchial balloon was associated with a better reduction in the risk of moderate bleeding and increased bronchoscopists’ confidence of sampling the lung tissue. Meanwhile, it may also reduce the incidence of severe bleeding. However, there was no effect on the improvement in diagnostic rate of MDD. Our study provided some level of evidence for clinical practice.

### Supplementary Information

**Additional file1: Figure S1.** Sample size calculated using PASS 11.

## Data Availability

All data generated or analyzed during this study are included in this published article and its Additional files.

## Abbreviations

TBLC: Transbronchial lung cryobiopsy
ILD: Interstitial lung disease
MDD: Multidisciplinary discussion
SLB: Surgical lung biopsy
HP: Hypersensitivity pneumonitis
IPF: Idiopathic pulmonary fibrosis
FVC: Forced vital capacity
DLCO: Diffusing capacity of the lung for carbon monoxide
CTD: Connective tissue disease
NISP: Non-specific interstitial pneumonia
DIP: Desquamative interstitial pneumonia
RB: Respiratory bronchiolitis
COP: Cryptogenic organizing pneumonia
