# Supplementary figures and images for: Assessment of a randomized controlled trial on the safety of pre-placing bronchial balloons in transbronchial lung cryobiopsy for diagnosing interstitial lung disease
Source: Eur J Med Res. 2024 May 3;29:268. doi: 10.1186/s40001-024-01871-y (PMC11067187; doi:10.1186/s40001-024-01871-y)

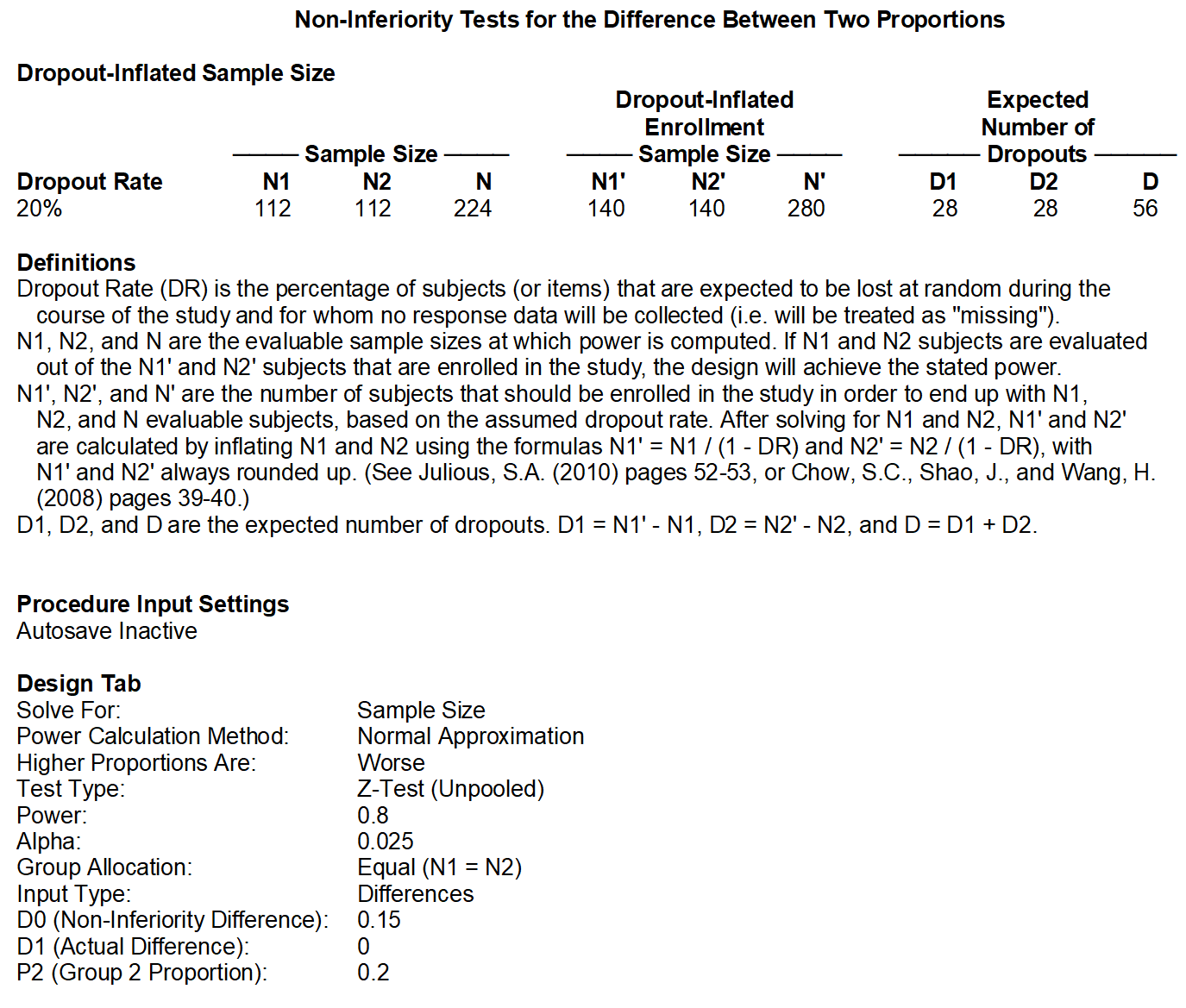


Figure S1. Sample size calculated using PASS 11

Supplement: Supplementary file 1 — Additional file1: Figure S1. Sample size calculated using PASS 11. [file 40001_2024_1871_MOESM1_ESM.docx]
